# Binary Hunter–Prey Optimization with Machine Learning—Based Cybersecurity Solution on Internet of Things Environment

**DOI:** 10.3390/s23167207

**Published:** 2023-08-16

**Authors:** Adil O. Khadidos, Zenah Mahmoud AlKubaisy, Alaa O. Khadidos, Khaled H. Alyoubi, Abdulrhman M. Alshareef, Mahmoud Ragab

**Affiliations:** 1 Information Technology Department, Faculty of Computing and Information Technology, King Abdulaziz University, Jeddah 21589, Saudi Arabia; 2 The Management of Digital Transformation and Innovation Systems in Organization Research Group, Faculty of Economics and Administration, King Abdulaziz University, Jeddah 21589, Saudi Arabia; 3 Department of Management Information System, Faculty of Economics and Administration, King Abdulaziz University, Jeddah 21589, Saudi Arabia; 4 Information Systems Department, Faculty of Computing and Information Technology, King Abdulaziz University, Jeddah 21589, Saudi Arabia; 5 Center of Research Excellence in Artificial Intelligence and Data Science, King Abdulaziz University, Jeddah 21589, Saudi Arabia; 6 Mathematics Department, Faculty of Science, Al-Azhar University, Naser City, Cairo 11884, Egypt

**Keywords:** Internet of Things, phishing attack, machine learning, hunter prey optimization, feature selection

## Abstract

Internet of Things (IoT) enables day-to-day objects to connect with the Internet and transmit and receive data for meaningful purposes. Recently, IoT has resulted in many revolutions in all sectors. Nonetheless, security risks to IoT networks and devices are persistently disruptive due to the growth of Internet technology. Phishing becomes a common threat to Internet users, where the attacker aims to fraudulently extract confidential data of the system or user by using websites, fictitious emails, etc. Due to the dramatic growth in IoT devices, hackers target IoT gadgets, including smart cars, security cameras, and so on, and perpetrate phishing attacks to gain control over the vulnerable device for malicious purposes. These scams have been increasing and advancing over the last few years. To resolve these problems, this paper presents a binary Hunter–prey optimization with a machine learning-based phishing attack detection (BHPO-MLPAD) method in the IoT environment. The BHPO-MLPAD technique can find phishing attacks through feature selection and classification. In the presented BHPO-MLPAD technique, the BHPO algorithm primarily chooses an optimal subset of features. The cascaded forward neural network (CFNN) model is employed for phishing attack detection. To adjust the parameter values of the CFNN model, the variable step fruit fly optimization (VFFO) algorithm is utilized. The performance assessment of the BHPO-MLPAD method takes place on the benchmark dataset. The results inferred the betterment of the BHPO-MLPAD technique over compared approaches in different evaluation measures.

## 1. Introduction

The Internet of Things (IoT) allows convergence and applications between real-time substances irrespective of their geographic localities [1]. Execution of these in network management and control makes the protection and privacy approach gain great importance and challenge in this setting [2]. IoT applications should protect data privacy from fixing security problems like jamming, intrusions, DoS attacks, eavesdropping, spoofing attacks, spam, malware, and DoS attacks [3]. The safety measure of IoT gadgets relies on the type and size of the entity in which it is enforced. The user’s behavior forces the security gateway to cooperate. In simple, the application, location, and nature of IoT gadgets decide the security measure [4]. For example, smart IoT security cameras can capture various variables for intellectual decision making and analysis in the smart organization [5]. The utmost care is to be taken with web-related gadgets, as more IoT gadgets depend on the web. It is ubiquitous in the workplace that the IoT gadgets deployed in an entity can be utilized for applying privacy and security features [6]. For instance, wearable gadgets that send and collect users’ health data to connected smartphones must avoid data leakage to ensure privacy. Nearly 25 to 30% of workforces link their personal IoT gadgets with the entity network [7]. The IoTs’ expanding nature allures the attackers and the users. 

The wide-ranging implementation of IoT gadgets by numerous entities, government sectors, trades, etc., is at high risk because of the devastating impact of data breaches and IoT gadget exploitation [8]. Hackers utilize the weakness of IoT gadgets, gain control over IoT gadgets, and then carry out malicious actions on confidential data with botnet attacks leading to the exposure of valuable information that causes financial loss [9]. One common threat that resulted in data breaches is phishing, a method where adversaries attempt to steal a user’s credentials utilizing fraud attempts [10]. Many large companies like Companies House (UK), Facebook, UPS, WhatsApp, and Fargo have experienced phishing attacks in recent years [11]. In addition to these phishing methods that use delicate data regarding their targets, phishing emails may be modified to look like real emails for increasing the response time to attacks [12]. There has been a rise in spear-phishing and email phishing attacks nowadays since these emails were aimed to directly attack victims, with an increased possibility of getting a response. Still, with the advent of ML in different attack scenarios [13], IoT devices select a protective approach and determine the critical parameter in the security protocol for a trade-off between computation, security, and privacy [14]. This is difficult since it is hard for an IoT platform with limited resources to predict the current network and prompt attack status [15].

The study introduces a binary Hunter–prey optimization with a machine learning-based phishing attack detection (BHPO-MLPAD) method in the IoT environment. The BHPO-MLPAD technique can detect phishing attacks through feature selection and classification. In the presented BHPO-MLPAD technique, the BHPO algorithm primarily chooses an optimal subset of features. The cascaded forward neural network (CFNN) model is employed for phishing attack detection. To adjust the parameter values of the CFNN model, the variable step fruit fly optimization (VFFO) algorithm is utilized. The performance assessment of the BHPO-MLPAD method takes place on the benchmark dataset. 

## 2. Literature Review

As IoT environments become increasingly susceptible to phishing threats, a comprehensive literature review is given to explore existing methodologies and advancements in phishing attack detection within this unique and complex ecosystem. Mughaid et al. [16] developed a detection method using an ML algorithm by splitting the data to train the recognition technique and validate the outcomes with the use of the testing dataset, to capture specific features of the emails and other characteristics to be categorized as phishing or non-phishing with three datasets, and we attained the most efficient and accurate outcomes after making a comparison between them. Abdulrahman et al. [17] introduced an effective ML-based method with the potential to find whether the website is phishing or not. Performance validation of the popular classification method was implemented and revealed Random Forest as the better classifier for the phishing data. An ML-based method for recognizing phishing attacks was constructed using RF with a wrapper based on the classifier attributes evaluator and ranker (CAER) feature selection model.

Jain and Gupta [18] introduced an ML-based anti-phishing technique (PHISH-SAFE) with URL features. We have considered fourteen features from URLs for detecting a web page as phishing or non-phishing to evaluate the performance of the presented method. The presented technique is trained by around 33,000 phishing and legitimate URLs with NB and SVM classifiers. Huang et al. [19] developed a new phishing website detection method by identifying the URL websites that is proved to be an efficient and robust detection method. Specifically, the new capsule-based NN primarily involves many related branches where a single convolution layer extracted shallow features from the URL, and the succeeding two capsule layers produce precise feature representation of the URL from shallow features and discriminates the legitimacy of the URL.

The author in [20] investigated an agreement on a definitive feature that must be utilized in phishing recognition. Fuzzy Rough Set (FRS) concept selects an efficient feature from three benchmarked datasets. The features selected are given to three commonly utilized classifiers for phishing recognition. Jain and Gupta [21] developed a method to identify phishing attacks in e-banking and commercial websites through the link and visual similarity. Phishers often try to stimulate the visual design of a website, and fake websites have hyperlinks and identify keywords that point towards legitimate webpages for trapping Internet users. Thus, the presented method inspects keywords, hyperlinks, and CSS layout of websites to identify phishing attacks. Azeez et al. [22] introduced an automatic whitelist method for recognizing phishing. The whitelist can be defined by implementing a thorough review between the actual and the visual links. The similarity of the known trusted websites can be evaluated with the content of the whitelist and matching it with the IP address beforehand, making decisions and inspecting the actual and visual links by evaluating the similarity of the known trusted website. In study [23], the authors devised an email phishing detection structure CNNPD, depending on CNN. CNNPD identify incoming emails as benign or phishing. 

In study [24], a novel MFO-RELM approach was presented for cyber-security threat detection and classification in the IoT platform. The proposed MFO-RELM approach achieves the effective detection of cybersecurity attacks that occur in the IoT platform. Ruiz-Villafranca et al. [25] examined MECInOT which is a structure dependent upon openLEON and able of creating test conditions for the IoT platform. The performance of this structure has been validated by generating an intelligent attack detector dependent upon tree-based algorithms, namely, RF, DT, and other ML approaches. Rookard and Khojandi [26] introduced a reinforcement learning-based network IDS for detecting attacks on IoT systems employing the TON-IoT database. Specially, the authors utilized the usage of DQN for cyber-attack detection. The authors defined that our DQN carries out an optimum for cyber-attack recognition. Mengash et al. [27] developed a novel search and rescue optimizer with ML-enabled cybersecurity method for an online social networks (SRO-MLCOSN) approach. The proposed SRO-MLCOSN approach concentrates on the detection of CB that ensued in social media.

The research gap exists in the scarcity of studies that systematically explore and optimize the highly related features specific to IoT data and the lack of comprehensive investigations into fine-tuning hyperparameters to achieve optimal performance for phishing detection in this unique and dynamic setting. Existing research often concentrates on traditional feature sets and generic hyperparameter settings, failing to address the IoT-specific challenges and intricacies that can significantly impact detection accuracy and robustness in real-world IoT scenarios. A more targeted and in-depth exploration of feature selection techniques and hyperparameter optimization tailored to the IoT environment is needed to enhance the effectiveness and reliability of phishing attack detection in IoT systems. Table 1 provides a summary of the existing works discussed in the literature.

## 3. The Proposed Model

**Figure 1:**
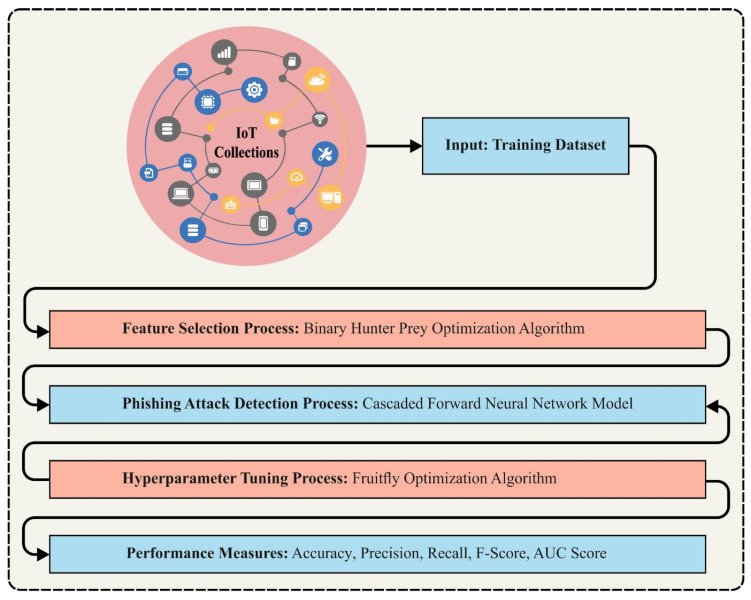
Workflow of BHPO-MLPAD approach.

This paper uses an automated phishing attack detection method, the BHPO-MLPAD technique, in the IoT environment. The BHPO-MLPAD technique can find phishing attacks through feature selection and classification. In the presented BHPO-MLPAD technique, a series of subprocesses are followed: BHPO-based feature subset selection, CFNN- based attack detection, and VFFO-related parameter tuning. Figure 1 depicts the workflow of the BHPO-MLPAD approach. 

### 3.1. BHPO-Based Feature Selection

Here, the BHPO algorithm primarily chooses an optimal subset of features and reduces the computation complexity. HPO is a newly developed metaheuristic approach to resolving the optimization problem [28]. This model is stimulated by predatory behavior between predator animals, like leopards, lions, and wolves, and prey, including gazelles, deer, and stags. The calculation method and principles are referred to as Naruei.

As per Naruei, the typical HPO technique performs better in resolving continuity issues but because of the uniqueness of discrete problems, the continuous HPO technique could not attain the best solutions. The “0–1” problem can be an integer programming problem, mathematically expressed below:

$$\max\;\;Z=\overset{D}{\underset{i=1}{\sum }}q_{i^{X}i}$$

(1) $$s.t.\;\overset{D}{\underset{i=1}{\sum }}\omega_{i}x_{i}<V,$$

$$x_{i}\in \left\{0,\;1\right\},\;i=1,2,\;\cdots ,\;D.$$

In Equation (1), D denotes the overall amount of items, $\chi_{i}$ indicates the i−th items chosen by the travelers, the respective weight is $\omega_{i}$, the value is $q_{i}$, and V signifies the maximal load.

Since the “0–1” problem restricts all the dimensions of the parameter to 0 or 1, it was not appropriate to apply the continuous method to resolve the problem; a binary discrete algorithm was used to resolve these problems. A binary HPO technique is developed that could efficiently resolve the ”0–1” issues making the typical HPO method inappropriate for resolving discreteness [29].

The generation model of the initial population is given below:

(2) $$x_{j}=\left\{\begin{array}{l} 1{\;\mathrm{if}\;R}_{1}>0.5\\ 0\;\mathrm{otherwise} \end{array}\;\right.$$

In Equation (2), $x_{i}$ shows the location of $i^{t}$s dimensions in all the individuals, and $R_{1}$ means the randomly generated value within [0–1]. The location of every individual’s dimensions in the population comprises 0 or 1 once the population is initialized. Whether this location is 0 or 1 is defined by the random value within [0, 1] produced by this location. When the randomly generated value is more extensive than 0.5, this location is 1; or else, this location is 0.The metaheuristics approach has different ways to expand the continuity model into a binary model; however, it is the most effective and easiest way to utilize the transfer function. A transfer function mapped the continuous real value of inputs to values within [0,1]. There are different types of transfer functions; here, we apply the more often used transformation function, that is, Sigmoid function:

(3) $$S\left(x\left(t+1\right)\right)=\frac{1}{1+e^{-x\left(t+1\right)}}$$

In Equation (3), $x\left(t+1\right)$ denotes the prey location or hunter for the following iteration. Even though the individual in the population was normalized through the transformation function, it is still essential to transform the mapped value from zero to one:

(4) $$x\left(t+1\right)=\left\{\begin{array}{ll} 0 & \mathrm{if}\;S\left(x\left(t+1\right)\right)\geq R_{2}\\ 1 & \mathrm{if}\;S\left(x\left(t+1\right)\right)<R_{2} \end{array}\right.\;$$

In Equation (4), $R_{2}$ denoted the randomly generated constant within [0, 1].

Once the typical HPO approach upgrades the location of prey or hunter, the binary solution is effectively attained by discrete processing [30]. The binary Hunter–prey optimization (BHPO) technique maintains the features of the typical HPO method. 

### 3.2. Phishing Attack Detection

**Figure 2:**
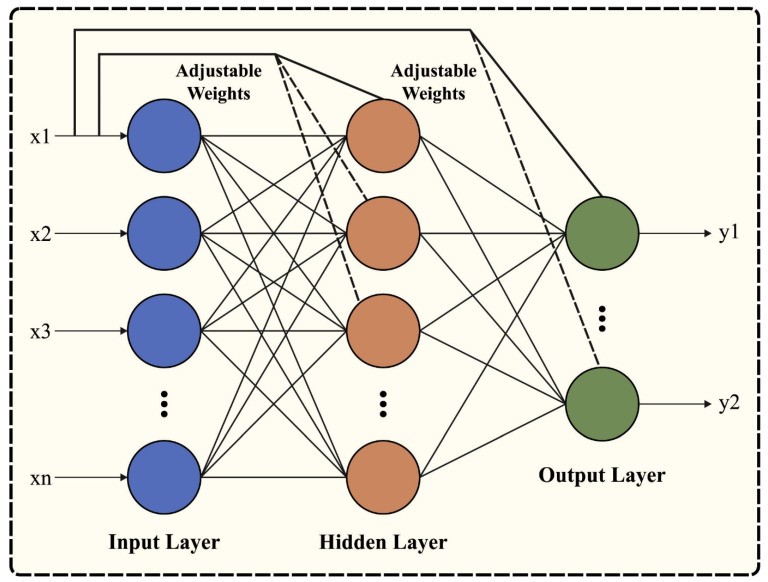
Architecture of CFNN.

At this stage, the phishing attack detection process is performed by the CFNN model. CFNN is a kind of NN that performs similarly to an FFNN. The major difference between FNN and CFNN is that it has a link with the prior HLs and input that provides the benefits of integrating the nonlinear relationships without eliminating the linear relationships between output and input [31]. Furthermore, it is a standard network since it needs fewer neurons to resolve the problems than FNN, making it efficient and compact. It includes hidden, input, and output layers. All the layers have different neurons and each layer is connected. Figure 2 illustrates the infrastructure of CFNN.

Utilizing the data from the input layer $\left(I_{i}\right)$, a weighted sum can be defined by a biased value $\left(b_{i}\right)$, and the summation function, that is commonly an endless number, is included to alter the outputs. The activation function $\left(f_{act}\right)$ was leveraged for transferring the weighted sum to the output value [32]. Here, activation functions are applied for output, and hidden layers are pure linear $\left(a_{2}\right)$ and tangent sigmoid (a), formulated as follows:

(5) $$a_{1}=\frac{1-e^{-2x}}{1+e^{-2x}}\;$$

$$a_{2}=x$$

The calculation at single hidden neuron (H) and output neuron (Out) are given below:

(6) $$H_{i}=f_{act}\left(\overset{m}{\underset{i=0}{\sum }}\left(I_{i}\times W_{ij}\right)+b_{i}\right)$$

(7) $$Out_{k}=f_{act}\left(\overset{n}{\underset{k=0}{\sum }}(H_{j}\times W_{jk}+I_{i}\times W_{ik})+b_{k}\right)$$

where $H_{j}$ denotes the hidden neuron, $W_{ij},$ $W_{\mathrm{jk}}$, and $W_{ik}$ represent the weight vector, and $b_{k}$ indicates the biased value.

### 3.3. VFFO-Based Parameter Tuning

Finally, the VFFO algorithm is used to adjust the parameter values of the CFNN model. The FFO algorithm is a recent approach to search for global optimization depending on foraging behaviors of FFs [33]. The optimization method is split into two stages. Firstly, the FF population exploits an olfactory search to discover the optimum solution, and later, other FFs exploit a visual search to determine the optimum individual and fly toward the direction. This can be repetitive until the fittest solution is found.

The primary steps of the FFO are given below:

Step 1: Randomly initialize the location of the FF population:

(8) $$Init\;X_{-axis},\;Init\;\gamma_{-axis}\;\;$$

Step 2: An FF performs a random search for generating a new location:

(9) $$\left\{\begin{array}{l} X_{i}=X_{-axis}+Random\;Value\\ \gamma_{i}=\gamma_{-axis}+Random\;Value \end{array}\right.$$

Step 3: Compute the distance between the origin and the individual FF and later attain the taste judgment value $S_{i}$:

(10) $$Disti=sqrt\left(X_{i}^{2}+\gamma_{i}^{2}\right)\;\;$$

(11) $$S_{i}=\frac{1}{Disti}\;\;$$

Step 4: The taste judgment values substituted with the judgment function for obtaining fitness) value of the FFs:

(12) $$Smell\left(i\right)=Function\left(S\right)$$

Step 5: Retain optimum fitness fruit fly:

(13) $$\left[bestSmell\;bestIndex\right]=\min\left(Smell\right)\;\;\;\;$$

Step 6: Record the fitness value and location of the better individuals. Next, each of the flies fly toward the location using a visual search:

(14) Smellbest=bestSmell  

(15) $$\left\{\begin{array}{l} X_{-axis}=X\left(bestIndex\right)\\ \gamma_{-axis}=\gamma \left(bestIndex\right) \end{array}\right.$$

Step 7: In an iterative operation, repeat steps 2 to 6; the optimum FF is output once the maximal iterative value is obtained.

The FFO algorithm has the lesser control parameter, usability, and simple structure, and its running speed was very fast [34]. But the FFO has related problems to other SI techniques. The optimization can be disorderly and blind, and the search range was smaller, which leads to local optimal solutions and lower optimization accuracy that are easier to fall into local optima because of the random search step sizes leveraged in the process of iterative optimization. In the VFFO method, a dynamic search step size was exploited to enhance the optimization method of the FFO in response to this deficiency, using the ordered convergence features of function to optimize the algorithm efficacy and balance the local optimization and global search abilities:

(16) $$l_{v}=e^{i/gen}-w\;*\;i\;*\;e^{-i/gen}$$

where i characterizes the existing FF individual, gen denotes the existing amount of iterations, and w shows the weight factor of 0 to 1. To explain the search curve, every generation of search steps has taken a minimal value. The population size was 50, and the maximal amount of iterations was 500 once the weight factor was fixed at 0.8. The variable step sizes enhance the range of search step sizes which change in the original model, considerably extending an efficient searching space of the model and enhancing a variety of solutions. Moreover, the search step size could attain a convergence rate with the rise in iteration, which makes the algorithm’s resolving procedure effective and orderly, efficiently enhancing the optimization performance and resolving the drawbacks of random search step size [35].

The fitness selection was a crucial factor in the VFFO approach. Solution encoding was utilized for assessing the goodness of solution candidate. The accuracy value was the major condition used to devise a fitness function:

(17) $$Fitness=\;\max\;\left(P\right)$$

(18) $$P=\frac{TP}{TP+FP}$$

where *FP* and *TP* indicate the false and true positive values.

## 4. Experimental Evaluation

The proposed model is simulated using the Python 3.6.5 tool. The outcomes of the BHPO-MLPAD technique can be investigated on the UNSW dataset [36], which holds 6000 samples and six classes, as provided in Table 2.

**Figure 3:**
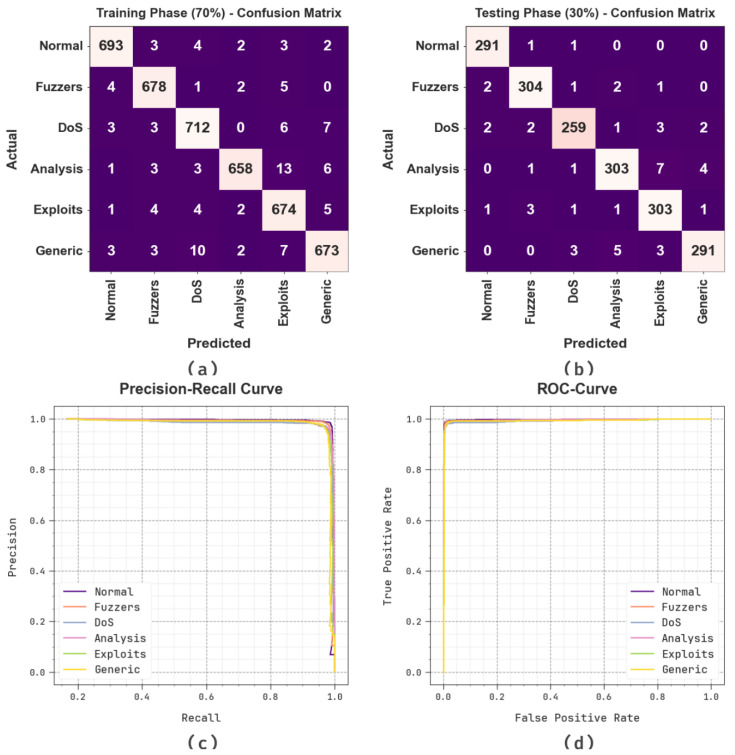
Classification outcome of (**a**,**b**) Confusion matrices, (**c**) PR-curve, and (**d**) ROC-curve.

Figure 3 exhibits the classifier results of the BHPO-MLPAD method under the test dataset. Figure 3a,b portrays the confusion matrix rendered by the BHPO-MLPAD approach on 70:30 of TRP/TSP. The result indicated that the BHPO-MLPAD algorithm has precisely classified and identified all six class labels. Likewise, Figure 3c reveals the PR analysis of the BHPO-MLPAD method. The figures stated that the BHPO-MLPAD methodology has gained maximal PR performance under six classes. Eventually, Figure 3d shows the ROC study of the BHPO-MLPAD method. The figure depicted that the BHPO-MLPAD algorithm has productive outcomes with higher ROC values under six class labels.

**Figure 4:**
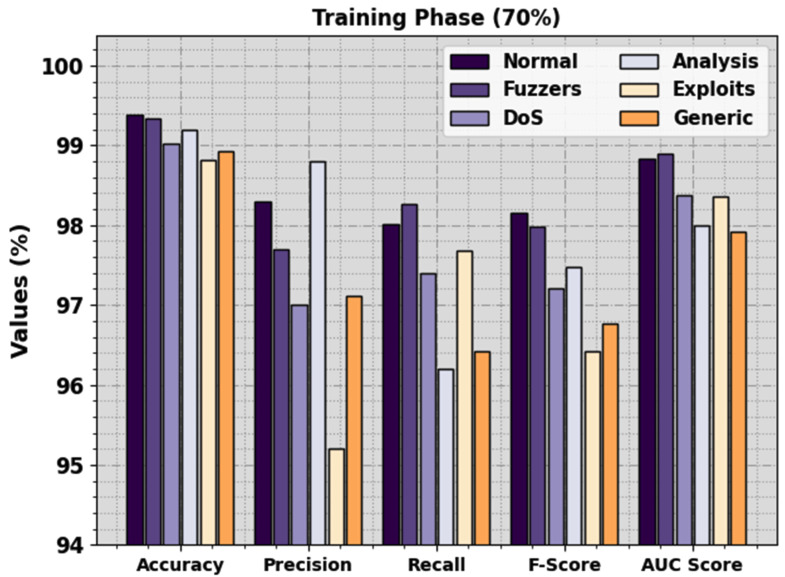
Detection outcome of the BHPO-MLPAD approach on 70% of TRP.

In Figure 4, the detection outcomes of the BHPO-MLPAD technique are clearly stated under 70% of TRP. The experimental outcomes highlighted that the BHPO-MLPAD technique recognized six types of classes. In the normal class, the BHPO-MLPAD technique attains $accu_{y}$ of 99.38%, $prec_{n}$ of 98.30%, $reca_{l}$ of 98.02%, $F_{score}$ of 98.16%, and $AUC_{score}$ of 98.84%. Also, in the Fuzzers class, the BHPO-MLPAD method reaches $accu_{y}$ of 99.33%, $prec_{n}$ of 97.69%, $reca_{l}$ of 98.26%, $F_{score}$ of 97.98%, and $AUC_{score}$ of 98.90%. Additionally, in the DoS class, the BHPO-MLPAD approach reaches $accu_{y}$ of 99.02%, $prec_{n}$ of 97%, $reca_{l}$ of 97.40%, $F_{score}$ of 97.20%, and $AUC_{score}$ of 98.38%. Lastly, in the Generic class, the BHPO-MLPAD algorithm achieves $accu_{y}$ of 98.93%, $prec_{n}$ of 97.11%, $reca_{l}$ of 96.42%, $F_{score}$ of 96.76%, and $AUC_{score}$ of 97.92%.

The overall performance of the BHPO-MLPAD technique is revealed in Table 3.

**Figure 5:**
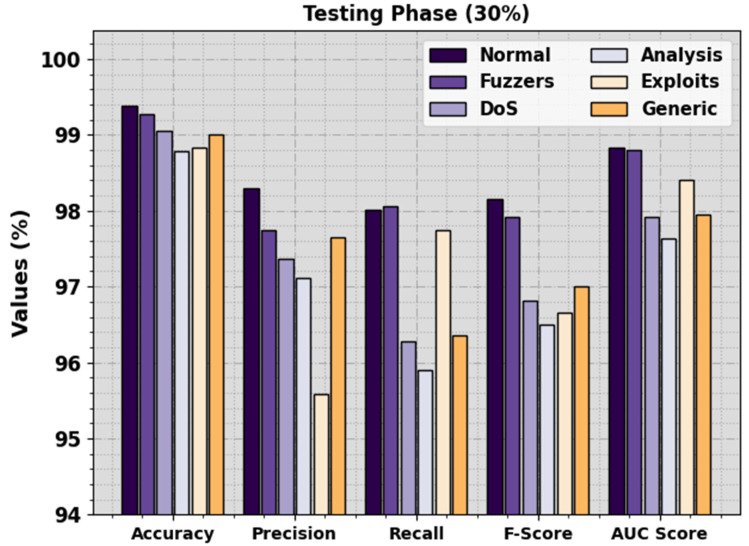
Detection outcome of BHPO-MLPAD approach on 30% of TSP.

In Figure 5, the detection outcomes of the BHPO-MLPAD method are clearly stated under 30% of TSP. The outcomes emphasized that the BHPO-MLPAD algorithm recognized six types of classes. In the normal class, the BHPO-MLPAD method reaches $an\;accu_{y}$ of 99.61%, $prec_{n}$ of 98.31%, $reca_{l}$ of 99.32%, $F_{score}$ of 98.81%, and $AUC_{score}$ of 99.49%. Similarly, in the Fuzzers class, the BHPO-MLPAD method attains $accu_{y}$ of 99.28%, $prec_{n}$ of 97.75%, $reca_{l}$ of 98.06%, $F_{score}$ of 97.91%, and $AUC_{score}$ of 98.80%. Furthermore, in the DoS class, the BHPO-MLPAD method attains $accu_{y}$ of 99.06%, $prec_{n}$ of 97.37%, $reca_{l}$ of 96.28%, $F_{score}$ of 96.82%, and $AUC_{score}$ of 97.91%. Lastly, in the Generic class, the BHPO-MLPAD approach attains an $accu_{y}$ of 99%, $prec_{n}$ of 97.65%, $reca_{l}$ of 96.36%, $F_{score}$ of 97%, and $AUC_{score}$ of 97.95%. 

**Figure 6:**
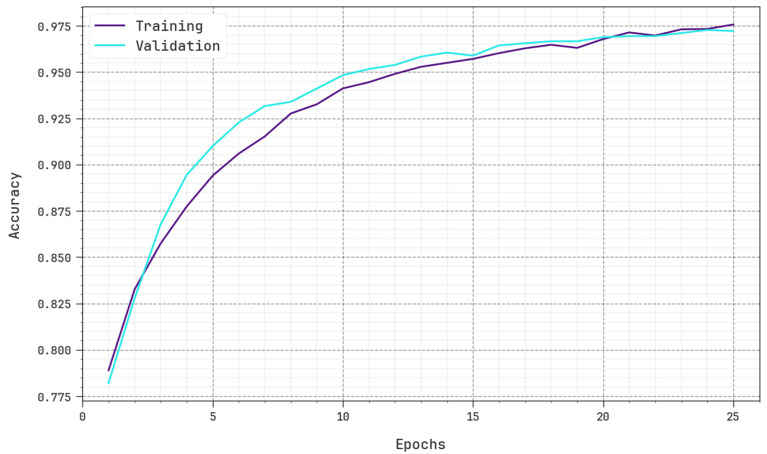
Accuracy curve of the BHPO-MLPAD approach.

Figure 6 inspects the accuracy of the BHPO-MLPAD method in the training and validation of the test database. The result specifies that the BHPO-MLPAD method reaches greater accuracy values over higher epochs. As well, the greater validation accuracy over training accuracy displays that the BHPO-MLPAD method learns productively on the test database.

**Figure 7:**
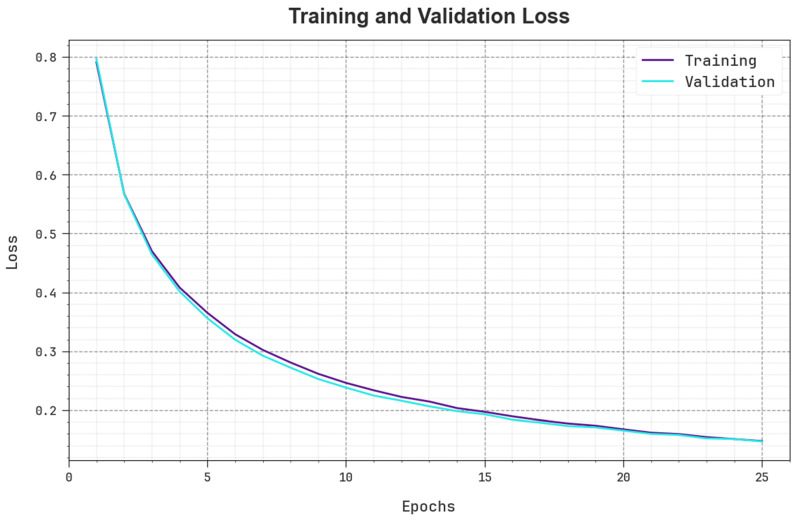
Loss curve of the BHPO-MLPAD approach.

The loss analysis of the BHPO-MLPAD method in training and validation is shown on the test database in Figure 7. The result indicates that the BHPO-MLPAD algorithm reaches adjacent training and validation loss values. The BHPO-MLPAD method learns productively on the test database.

**Figure 8:**
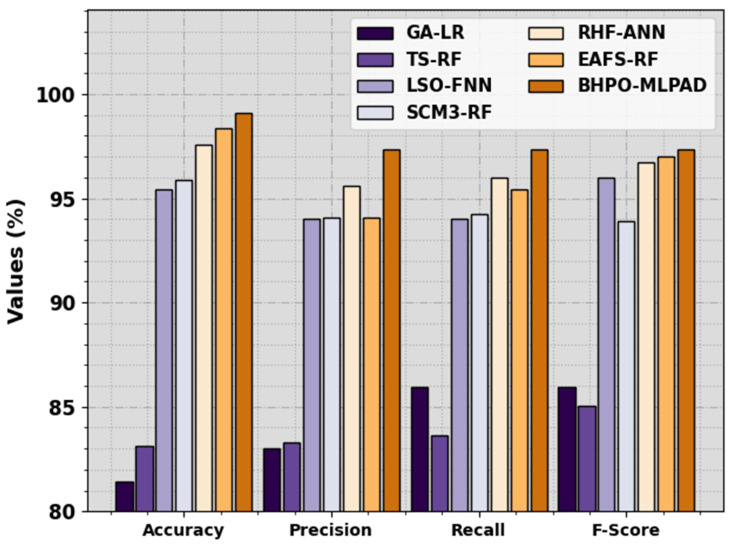
Comparative outcome of the BHPO-MLPAD approach with other methodologies.

A detailed comparative result of the BHPO-MLPAD technique is reported in Table 4 and Figure 8. The results stated that the GA-LR and TS-RF models have revealed worse results over other models.

Along with the aforementioned, the LSO-FNN and SCM3-RF models have obtained poor performance. On the contrary, the RHF-ANN and EAFS-RF models attained slightly improved results. However, the BHPO-MLPAD technique stated the maximum performance of the BHPO-MLPAD technique over other models with $accu_{y}$ of 99.11%, $prec_{n}$ of 97.35%, $reca_{l}$ of 97.33%, and $F_{score}$ of 97.33%.

**Figure 9:**
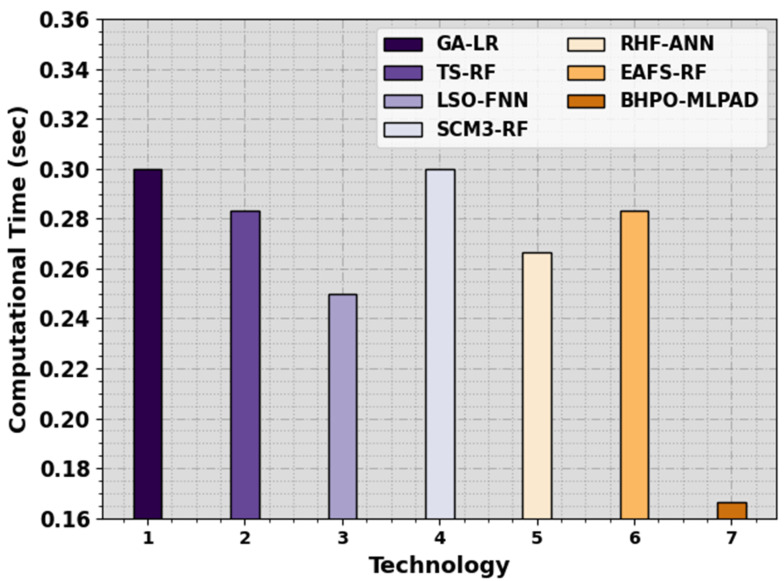
CT outcome of the BHPO-MLPAD approach with other methodologies.

Finally, the brief computation time (CT) results of the BHPO-MLPAD method are compared with other models in Table 5 and Figure 9. The results showed that the BHPO-MLPAD technique accomplished the least CT of 0.17 s. On the contrary, the existing models such as GA-LR, TS-RF, LSO-FNN, SCM3-RF, RHF-ANN, and EAFS-RF models have obtained increased CT values of 0.30 s, 0.28 s, 0.25 s, 0.30 s, 0.27 s, and 0.28 s, respectively. These results highlighted that the BHPO-MLPAD technique achieved better performance over other models in the IoT environment. 

## 5. Conclusions

In this paper, an automated phishing attack detection technique, named BHPO-MLPAD technique, has been used in the IoT environment. The BHPO-MLPAD technique is able to detect phishing attacks through feature selection and classification. In the presented BHPO-MLPAD technique, a series of subprocesses are followed: BHPO-based feature subset selection, CFNN-based attack detection, and VFFO-based parameter tuning. Here, the BHPO algorithm primarily chooses an optimal subset of features and reduces the computation complexity. Next, the phishing attack detection process is performed by the CFNN method. Finally, the VFFO algorithm is utilized to adjust the parameter values of the CFNN method. The performance assessment of the BHPO-MLPAD method takes place on the benchmark dataset. The outcomes inferred the betterment of the BHPO-MLPAD method over compared approaches in terms of various evaluation measures.

## Figures and Tables

**Table 1:** Summary of existing works.

Reference	Year	Method	Performance	Dataset
Mughaid et al. [16]	2022	ML models such as SVM, DT, LR, NN, and decision forest	Accuracy, Precision, Recall, F-Score	Phishing email collection dataset
Abdulrahman et al. [17]	2019	Random Forest and CAER feature selection	TPR, FPR, Accuracy, Precision, Recall, F-Measure	UCI phishing website dataset
Jain et al. [18]	2018	PHISH-SAFE, an ML-based classifier	Accuracy	PhishTank URL dataset
Huang et al. [19]	2019	Capsule-based neural network	TPR, FPR, Accuracy, Precision, Recall, F-Measure	PhishTank and Openphish data
Zabihimayvan and Doran [20]	2019	Fuzzy Rough Set	F-measure	UCI Phishing and Mendeley dataset
Jain and Gupta [21]	2018	Link and visual similarity relation	TPR, FPR	-
Azeez et al. [22]	2021	Whitelist approach	TPR, TNR, FPR, FNR, Accuracy	PhishTank and Alexa data
Alotaibi et al. [23]	2020	CNN	Accuracy	PhishingCorpus and SpamAssassin
Alrowais et al. [24]	2023	Mayfly optimization with RELM	Accuracy, Precision, Recall, F-score	N-BaIoT dataset
Ruiz-Villafranca et al. [25]	2023	MECInOT	Accuracy, Precision, Recall, F-score	Mendeley dataset
Rookard and Khojandi [26]	2023	Deep Q-network	Accuracy	-
Mengash et al. [27]	2023	SRO-MLCOSN model	Accuracy, Precision, Recall, F-score	-

**Table 2:** Details of the dataset.

Class	No. of Samples
Normal	1000
Fuzzers	1000
DoS	1000
Analysis	1000
Exploits	1000
Generic	1000
Total Number of Samples	6000

**Table 3:** Detection outcome of the BHPO-MLPAD approach on 70:30 of TRP/TSP.

Class	$Accu_{y}$	$Prec_{n}$	$Reca_{l}$	$F_{score}$	$AUC_{score}$
Training Phase (70%)					
Normal	99.38	98.30	98.02	98.16	98.84
Fuzzers	99.33	97.69	98.26	97.98	98.90
DoS	99.02	97.00	97.40	97.20	98.38
Analysis	99.19	98.80	96.20	97.48	97.99
Exploits	98.81	95.20	97.68	96.42	98.36
Generic	98.93	97.11	96.42	96.76	97.92
Average	99.11	97.35	97.33	97.33	98.40
Testing Phase (30%)					
Normal	99.61	98.31	99.32	98.81	99.49
Fuzzers	99.28	97.75	98.06	97.91	98.80
DoS	99.06	97.37	96.28	96.82	97.91
Analysis	98.78	97.12	95.89	96.50	97.64
Exploits	98.83	95.58	97.74	96.65	98.40
Generic	99.00	97.65	96.36	97.00	97.95
Average	99.09	97.30	97.28	97.28	98.36

**Table 4:** Comparative outcome of the BHPO-MLPAD approach with other methodologies.

Technology	$Accu_{y}$	$Prec_{n}$	$Reca_{l}$	$F_{score}$
GA-LR	81.42	83.03	85.93	85.95
TS-RF	83.12	83.28	83.63	85.06
LSO-FNN	95.42	94.03	94	95.98
SCM3-RF	95.87	94.08	94.22	93.89
RHF-ANN	97.60	95.62	95.98	96.71
EAFS-RF	98.36	94.08	95.41	97.01
BHPO-MLPAD	99.11	97.35	97.33	97.33

**Table 5:** CT outcome of the BHPO-MLPAD approach with other methodologies.

Technology	Computational Time (s)
GA-LR	0.30
TS-RF	0.28
LSO-FNN	0.25
SCM3-RF	0.30
RHF-ANN	0.27
EAFS-RF	0.28
BHPO-MLPAD	0.17

## Data Availability

Data sharing is not applicable to this article as no datasets were generated during the current study.

## References

[1] Ansari M.F., Sharma P.K., Dash B. (2022). Prevention of phishing attacks using AI-based Cybersecurity Awareness Training. Prevention.

[2] Subramanian S., Venkatachalam N., Rajendran R. (2023). A Novel Phishing Attack Prediction Model With Crowdsouring in Wireless Networks. Perspectives on Social Welfare Applications’ Optimization and Enhanced Computer Applications.

[3] Basit A., Zafar M., Liu X., Javed A.R., Jalil Z., Kifayat K. (2021). A comprehensive survey of AI-enabled phishing attacks detection techniques. Telecommun. Syst..

[4] Andryukhin A.A. Phishing attacks and preventions in blockchain based projects. Proceedings of the 2019 International Conference on Engineering Technologies and Computer Science (EnT).

[5] Abu Al-Haija Q., Zein-Sabatto S. (2020). An efficient deep-learning-based detection and classification system for cyber-attacks in IoT communication networks. Electronics.

[6] Basheri M., Ragab M. (2023). Quantum Cat Swarm Optimization Based Clustering with Intrusion Detection Technique for Future Internet of Things Environment. Comput. Syst. Sci. Eng..

[7] Elsisi M., Tran M.Q., Mahmoud K., Mansour D.E.A., Lehtonen M., Darwish M.M. (2021). Towards secured online monitoring for digitalized GIS against cyber-attacks based on IoT and machine learning. IEEE Access.

[8] Sivanathan A., Gharakheili H.H., Sivaraman V. (2020). Managing IoT cyber-security using programmable telemetry and machine learning. IEEE Trans. Netw. Serv. Manag..

[9] Panda M., Abd Allah A.M., Hassanien A.E. (2021). Developing an efficient feature engineering and machine learning model for detecting IoT-botnet cyber attacks. IEEE Access.

[10] Alam M.N., Sarma D., Lima F.F., Saha I., Hossain S. (2020). August. Phishing attacks detection using machine learning approach. In Proceedings of the 2020 Third International Conference on Smart Systems and Inventive Technology (ICSSIT).

[11] Espinoza B., Simba J., Fuertes W., Benavides E., Andrade R., Toulkeridis T. (2019). December. Phishing attack detection: A solution based on the typical machine learning modeling cycle. In Proceedings of the 2019 International Conference on Computational Science and Computational Intelligence (CSCI).

[12] Gupta B.B., Jain A.K. (2020). Phishing attack detection using a search engine and heuristics-based technique. J. Inf. Technol. Res..

[13] Demertzis K., Iliadis L. (2019). Cognitive web application firewall to critical infrastructures protection from phishing attacks. J. Comput. Model..

[14] Alsariera Y.A., Adeyemo V.E., Balogun A.O., Alazzawi A.K. (2020). Ai meta-learners and extra-trees algorithm for the detection of phishing websites. IEEE Access.

[15] Alsufyani A.A., Alzahrani S.M. (2021). Social Engineering Attack Detection Using Machine Learning: Text Phishing Attack. Indian J. Comput. Sci. Eng..

[16] Mughaid A., AlZu’bi S., Hnaif A., Taamneh S., Alnajjar A., Elsoud E.A. (2022). An intelligent cyber security phishing detection system using deep learning techniques. Clust. Comput..

[17] Abdulrahman M.D., Alhassan J.K., Adebayo O.S., Ojeniyi J.A., Olalere M. (2019). Phishing attack detection based on random forest with wrapper feature selection method. Int. J. Inf. Process. Commun..

[18] Jain A.K., Gupta B.B. (2018). PHISH-SAFE: URL features-based phishing detection system using machine learning. Cyber Security: Proceedings of CSI 2015.

[19] Huang Y., Qin J., Wen W. (2019). Phishing URL detection via capsule-based neural network. Proceedings of the 2019 IEEE 13th International Conference on Anti-Counterfeiting, Security, and Identification (ASID).

[20] Zabihimayvan M., Doran D. (2019). Fuzzy rough set feature selection to enhance phishing attack detection. Proceedings of the 2019 IEEE International Conference on Fuzzy Systems (FUZZ-IEEE).

[21] Jain A.K., Gupta B.B. (2018). Detection of phishing attacks in financial and e-banking websites using link and visual similarity relation. Int. J. Inf. Comput. Secur..

[22] Azeez N.A., Misra S., Margaret I.A., Fernandez-Sanz L. (2021). Adopting automated whitelist approach for detecting phishing attacks. Comput. Secur..

[23] Alotaibi R., Al-Turaiki I., Alakeel F. (2020). Mitigating email phishing attacks using convolutional neural networks. Proceedings of the 2020 3rd International Conference on Computer Applications & Information Security (ICCAIS).

[24] Alrowais F., Althahabi S., Alotaibi S.S., Mohamed A., Hamza M.A., Marzouk R. (2023). Automated machine learning enabled cyber security threat detection in Internet of things environment. Comput. Syst. Sci. Eng..

[25] Ruiz-Villafranca S., Carrillo-Mondéjar J., Castelo Gómez J.M., Roldán-Gómez J. (2023). MECInOT: A multi-access edge computing and industrial internet of things emulator for the modelling and study of cybersecurity threats. J. Supercomput..

[26] Rookard C., Khojandi A. (2023). Applying Deep Reinforcement Learning for Detection of Internet-of-Things Cyber Attacks. Proceedings of the 2023 IEEE 13th Annual Computing and Communication Workshop and Conference (CCWC).

[27] Mengash H.A., Alzahrani J.S., Eltahir M.M., Al-Wesabi F.N., Mohamed A., Hamza M.A., Marzouk R. (2023). Search and Rescue Optimization with Machine Learning Enabled Cybersecurity Model. Comput. Syst. Sci. Eng..

[28] Zhao Z., Rui Y., Liu Y., Liu Z., Tu Z. (2023). Topology Optimization of Continuum Structures Based on Binary Hunter-Prey Optimization Algorithm. Symmetry.

[29] Naruei I., Keynia F., Sabbagh Molahosseini A. (2022). Hunter–prey optimization: Algorithm and applications. Soft Comput..

[30] AbdelAty A.M., Yousri D., Chelloug S., Alduailij M., Abd Elaziz M. (2023). Fractional order adaptive hunter-prey optimizer for feature selection. Alex. Eng. J..

[31] Imran M., Khushnood R.A., Fawad M. (2023). A hybrid data-driven and metaheuristic optimization approach for the compressive strength prediction of high-performance concrete. Case Stud. Constr. Mater..

[32] Alkhasawneh M.S., Tay L.T. (2018). A hybrid intelligent system integrating the cascade forward neural network with elman neural network. Arab. J. Sci. Eng..

[33] Huang H., Tao D., Wei X., Zhou Y. (2023). Adaptive Image Enhancement Algorithm Based on Variable Step Fruit Fly Optimization Algorithm and Nonlinear Beta Transform. Biomimetics.

[34] Sun H., Li W., Zheng L., Ling S., Fu W. (2022). Adaptive co-simulation method and platform application of drive mechanism based on Fruit Fly Optimization Algorithm. Prog. Nucl. Energy.

[35] Du T.S., Ke X.T., Liao J.G., Shen Y.J. (2018). DSLC-FOA: Improved fruit fly optimization algorithm for application to structural engineering design optimization problems. Appl. Math. Model..

[36] Moustafa N., Jill S. (2015). UNSW-NB15: A comprehensive data set for network intrusion detection systems (UNSW-NB15 network data set). Proceedings of the Military Communications and Information Systems Conference (MilCIS).

